# The effect of stress on the expression of the amyloid precursor protein in rat brain

**DOI:** 10.1016/j.neulet.2007.11.032

**Published:** 2008-02-06

**Authors:** Rachel Sayer, Deborah Robertson, David J.K. Balfour, Kieran C. Breen, Caroline A. Stewart

**Affiliations:** Alzheimer's Disease Research Centre, Section of Psychiatry and Behavioural Sciences, Division of Pathology and Neuroscience, University of Dundee, Ninewells Hospital & Medical School, Dundee DD1 9SY, UK

**Keywords:** Elevated platform, Habituation, Amyloid, Alzheimer's disease, Hippocampus, Glycosylation

## Abstract

The abnormal processing of the amyloid precursor protein (APP) is a pivotal event in the development of the unique pathology that defines Alzheimer's disease (AD). Stress, and the associated increase in corticosteroids, appear to accelerate brain ageing and may increase vulnerability to Alzheimer's disease via altered APP processing. In this study, rats were repeatedly exposed to an unavoidable stressor, an open elevated platform. Previous studies in this laboratory have shown that a single exposure produces a marked increase in plasma corticosterone levels but animals develop tolerance to this effect between 10 and 20 daily sessions. Twenty-four hours after stress, there was an increase in the ratio of the deglycosylated form of APP in the particulate fraction of the brain, which subsequently habituated after 20 days. The levels of soluble APP (APPs) tended to be lower in the stress groups compared to controls except for a significant increase in the hippocampus after 20 days of platform exposure. Since APPs is reported to have neurotrophic properties, this increased release may represent a neuroprotective response to repeated stress. It is possible that the ability to mount this response decreases with age thus increasing the vulnerability to stress-induced AD-related pathology.

The role of amyloid precursor protein (APP), or its processed fragments, in normal brain function is not well understood. There is a significant body of evidence to suggest that the membrane-bound protein plays a role in cell–cell interaction, neuronal plasticity and the formation and the consolidation of synaptic connections [9]. Soluble APPα (APPsα) generated following cleavage by the α-secretase enzyme is thought to promote cell survival, neurite outgrowth and synaptogenesis [15]. The administration of APPsα in vivo can reduce the level of neuronal damage following traumatic brain injury [23] and increase the number of EGF-responsive progenitor cells [6]. Lower levels of APPs in rats were associated with poor performance in the watermaze [1] whereas intracerebroventricular administration of sAPP enhanced the performance of mice in various learning tasks [17]. However if membrane-bound APP is cleaved by the β- and γ-secretase protease enzymes it generates the Aβ peptide that aggregates to form the neuritic plaques which define Alzheimer's disease pathology [9].

For a majority of AD cases it is not known what causes the shift to the amyloidogenic pathway although there is some preclinical and clinical evidence to suggest that stress may exacerbate the progression of AD and may be associated with the pathological hallmarks of the condition. In animals, exposure to isolation stress accelerated the age-dependent deposition of amyloid plaques in the Tg2576 mouse model of AD [11]. Chronic immobilisation stress had a similar effect on both memory impairment and amyloid deposition within the hippocampus and cortex of the APP_V7171_-CT100 mouse model [13]. These effects may be a direct consequence of the corticosteroids released during the response to stress as prolonged exposure to high levels of these hormones appears to accelerate age-related decrements in neuronal morphology and function [19]. Studies on patients with AD have shown that hypercortisolism is associated with greater cognitive deficits [25] and that increased plasma cortisol is associated with greater decline in both clinical and cognitive measures of dementia severity [10].

The aim of this study was to determine whether exposure to acute and repeated stress affected the levels of the two primary forms of APP in the normal rat brain and whether this was regionally selective.

Male Sprague Dawley rats (Harlan, UK) weighing between 250 and 300 g were maintained on a 12:12 h light:dark cycle with lights on at 6.00 a.m. and access to food and water *ad libitum*. After 1 week they were divided into 4 treatment groups (*n* = 5 rats per group): a 20 days stress group, a 10 days stress group, an acute stress group (1 day) and a control group. Stress exposure involved placing the rats on an open elevated platform for 60 min. Acutely this procedure reliably increases the levels of corticosteroid hormones in the blood [2] but this response habituates after between 10 and 20 days exposure [18,22]. Control animals were not exposed to the stressor but were taken to the same laboratory and handled in an equivalent manner. All experimental procedures were subjected to local ethical review and covered by a U.K. Home Office project licence (PPL 60/2845).

Rats were killed 24 h after the last stress exposure or handling session to measure the effect of stress-induced genomic changes on APP. Brains were removed and the frontal cortex, parietal cortex, striatum and hippocampus were dissected on ice and stored at −80 °C prior to analysis. The pathological changes that characterise Alzheimer's disease first appear in the cortical regions of the brain and the hippocampus. The striatum was included as an area also rich in cholinergic neurones that remains relatively free of pathology until advanced stages of the disease. The brain samples were then homogenised (10% w/v) in homogenising buffer (1 mM EDTA, 1 mM EGTA, 0.32 M sucrose, 1 mM Tris, pH 8.0 containing protease inhibitor cocktail) at 4 °C. Homogenates were centrifuged at 100,000 × *g* for 60 min at 4 °C and the resulting supernatant, containing the soluble brain fraction, was aliquoted into 1 ml fractions and stored at −20 °C. The pellet was resuspended in 1% (v/v) Triton X-100 and re-centrifuged at 100,000 × *g* for 60 min at 4 °C. The supernatant, containing the membrane, mitochondrial and nuclear fractions, was stored at −20 °C in 1.0 ml aliquots while the triton-insoluble pellet was discarded.

The total protein concentration of each sample was determined spectrophotometrically using the method of Bradford [4] and APP expression determined in 25 ug protein samples by Western blot analysis using a monoclonal primary antibody (MAB348 clone 22C11 Calibochem 1:1500) [16]. This recognises an N-terminal epitope (AA66-81) that is common to both APP and the amyloid precursor-like protein 2 (APLP2). The autoradiographs were scanned and band densities calculated using the SCION image (v3) software package. In order to determine whether there were any regional differences in APP expression between the groups the data were analysed by repeated measures analysis of variance using stress exposure as the between subject factor (GROUP) and brain region as the within subject factor (REGION). Post-hoc analysis of significant effects was carried out using one-way ANOVA or Dunnett *t*-tests where appropriate.

In the soluble brain fractions APPs was detected as a single band with a molecular weight of approximately 128 kDa (Fig. 1). The global analysis revealed that there was a significant effect of GROUP (*F*[3, 16] = 3.79, *p* < 0.05) and a significant interaction between GROUP and REGION (*F*[9, 48] = 3.58, *p* < 0.005) suggesting that stress had a regionally selective effect on the level of APPs. Subsequent analysis of this interaction revealed that this was due to the significant increase in APPs in the hippocampal region only following 20 days of stress (*p* < 0.05).

In the particulate brain fractions, APP was detected as a doublet with molecular weights of approximately 128 and 121 kDa (Fig. 2A). Global analysis of total APP revealed a significant effect of GROUP (*F*[3, 16] = 8.02, *p* < 0.005) but no interaction between GROUP and REGION suggesting that stress had influenced the level of APP but that this was not a regionally selective effect. Post-hoc testing confirmed that all stress groups expressed a significantly greater level of APP protein in the particulate brain fractions compared to controls (*p* < 0.05). Therefore, although the pattern appeared to be different in the parietal cortex compared to other regions, this effect was not large enough to influence the overall statistical analysis.

The 121 kDa molecular weight protein band has previously been demonstrated to represent the deglycosylated form of the protein [16]. When this band was expressed as a percentage of total APP (Fig. 2B) global analysis confirmed a significant effect of GROUP (*F*[3, 16] = 11.6, *p* < 0.001) but again no interaction between GROUP and REGION suggesting that stress had affected the APP band ratio but not in a regionally selective way. Post-hoc testing confirmed that, overall, only the acute and 10 days stress group had a significantly increased proportion of deglycosylated APP relative to control (*p* < 0.05). Close inspection of the data (Fig. 2B) suggests that the APP band ratio was still increased above control levels in the frontal cortex after 20 days stress but this effect did not significantly influence the global analysis.

Although stress exposure appears to have altered the processing of APP with changes in the glycosylation patterns of the particulate form and the levels of APPs, it is not clear whether this is due to changes in the release of corticosterone over this time period [18,22] or if it is due to the action of other neurotransmitters, such as acetylcholine, that are altered following stress [14]. Other studies have shown that directly treating rats with the glucocorticoid agonist dexamethasone can also increase particulate APP levels in the rat cortex, cerebellum and brain stem without significant changes in APPs [5]. However, hippocampal APP expression was not specifically reported in that study. In this study, stress significantly elevated the proportion of deglycosylated APP in particulate brain samples for up to 10 days but after 20 days there was no significant difference from control. This follows the previously published pattern of habituation of the plasma corticosterone response in rats treated with exactly the same protocol [18,22]. Corticosteroids have also been shown to control the process of protein glycosylation and in the hippocampus sialyltranferase activity appears to be highly regulated by aldosterone [8]. The expression of the mineralocorticoid receptor, which has a relatively high affinity for aldosterone, is dynamically altered in the dorsal hippocampus during 20 days exposure to the elevated platform [18]. The stress-induced increase in the particulate deglycosylated form of the protein may therefore be a consequence of corticosteroid receptor changes. Although statistically, there was no significant difference between the ratio of deglycosylated to total APP in the hippocampus compared to other brain regions, this was the area that demonstrated the greatest change following acute exposure and was the only one where the ratio fell below control levels after 20 days of stress (Fig. 2B).

The hippocampus was also the only area where the amount of APPs increased following repeated stress. The hippocampus is involved in terminating the corticosteroid response to stress [20] and may also be important in habituating to repeated stress as previous studies have shown that 20 days exposure to the elevated platform produces a selective increase in GR immunoreactivity in the dorsal hippocampus [18]. The increase in APPs most likely represents an increase in soluble APPsα that has been shown to exhibit neurotrophic properties. Since the hippocampus it also an area of the brain thought to be crucial for the formation of certain types of memory, the changes in APPs may have consequences for cognitive function. Previous studies have shown that stress can impair memory [21] or under certain circumstances can enhance it [3]. In particular, repeated exposure to the elevated platform has been shown to improve spatial memory using a watermaze task in young rats but only if they have been previously exposed to the apparatus [26].

The increase in deglycosylated APP seen following one or 10 days stress exposure may indicate an increased risk of Aβ formation as the deglycosylated form of the protein is less likely to be trafficked to the cell membrane [16] for cleavage by α-secretase but rather may be retained in the endoplasmic reticulum/golgi. Since β-secretase appears to be localised to the golgi and endosomal compartments of the cell [24] the deglycosylated APP is more likely to undergo amyloidogenic processing by β- and γ-secretase pathway. Recent studies have shown that glucocorticoid treatment of either mouse neuronal cells or the 3XTg-AD mouse increases the formation of Aβ [12]. This appears to be due to an increase in the expression levels of both APP and β-secretase. The formation of Aβ peptide oligomers may also disrupt cognitive function, even prior to nerve cell death [7]. The increase in APPs within the hippocampus over the same period as habituation to a repeated stressor could therefore represent a compensatory response in relatively young adult rats. Further studies will be required to determine the exact mechanisms responsible for the changes in APP processing and whether an age-induced impairment in this neuroadaptation increases the risk of the stimulation of the AD-associated protein processing pathways.

## Figures and Tables

**Fig. 1:**
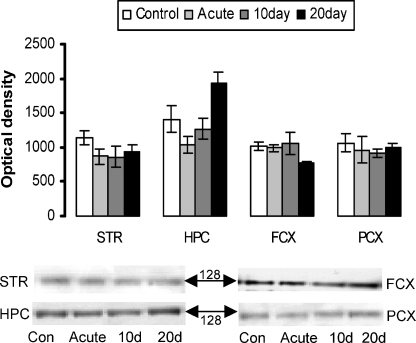
APP protein expression in soluble brain fractions. Striatum (STR), hippocampus (HPC), frontal cortex (FCX) and parietal cortex (PCX) generated from control rats and rats exposed to the elevated platform for 1 day (acute), 10 and 20 days. Values represent mean ± S.E.M. (*n* = 5 in each group). Representative blots are shown with the molecular weight of the protein bands indicated.

**Fig. 2:**
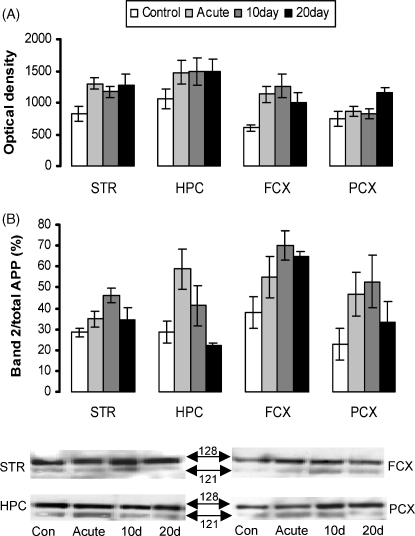
APP protein expression in particulate brain fractions. Striatum (STR), hippocampus (HPC), frontal cortex (FCX) and parietal cortex (PCX) generated from control rats and rats stressed on an elevated platform for 1 day (acute), 10 and 20 days. (A) Total APP protein expression in the particulate brain fractions. (B) The lower molecular weight band of the particulate form of APP (121 kDa) expressed as a percentage of total particulate APP levels. Representative blots are shown with the molecular weights of the protein bands indicated.
